# Biological approach synthesis and characterization of iron sulfide (FeS) thin films from banana peel extract for contamination of environmental remediation

**DOI:** 10.1038/s41598-022-14828-0

**Published:** 2022-06-21

**Authors:** Abel Saka, Leta Tesfaye Jule, Shuma Soressa, Lamessa Gudata, N. Nagaprasad, Venkatesh Seenivasan, Krishnaraj Ramaswamy

**Affiliations:** 1 Department of Physics, College of Natural and Computational Science, Dambi Dollo University, Dambi Dollo, Ethiopia; 2 Centre for Excellence in Technology Transfer and Incubation, Dambi Dollo University, Dambi Dollo, Ethiopia; 3 Department of Animal Science, College of Agriculture and Veterinary Medicine, Dambi Dollo University, Dambi Dollo, Ethiopia; 4 Department of Mechanical Engineering, ULTRA College of Engineering and Technology, Madurai, Tamil Nadu 625 104 India; 5 Department of Mechanical Engineering, Sri Eshwar College of Engineering, Coimbatore, India; 6 Mechanical Engineering Department, College of Engineering Science, Dambi Dollo University, Dambi Dollo, Ethiopia

**Keywords:** Biochemistry, Biological techniques, Drug discovery, Climate sciences, Environmental sciences, Environmental social sciences, Natural hazards, Medical research, Engineering, Materials science, Nanoscience and technology, Physics

## Abstract

Biological approach synthesis and characterization of Iron Sulfide (FeS) thin films from banana peel extract for contamination remediation of environment studied. Iron chloride, Sodium thiosulfate and Ethylene-di-amine-tetra acetate (EDTA) were used as precursor solutions without further purification. The nanoparticle of banana peel was extracted and prepared with synthesized FeS thin films and analyzed by X ray-diffraction for structural examination, Scanning electron microscope (SEM) for surface morphological analysis, Ultra-violet-visible-spectrometer (UV–Vis) and photo-luminescence spectro-photo-meter (P-L) for optical characterizations. XRD peaks are shown with recognized to (110), (200), (310), and (301) crystalline planes. The occurrence of this deflection peak are recognised the FeS crystal segment of the tetragonal crystalline systems. SEM micrographs of the films prepared biological method show the distribution of grains, which cover the surface of the substrate completely and are uniform and films deposited purely have defects. The photo-luminescence, absorbance, and transmittance strength of banana peel extract FeS thin film is greater than pure FeS thin films in which wide-ranging and symmetries groups were perceived. In the present study, the comparison of pure FeS thin films and Nano synthesized banana peel extract with FeS thin films was studied. It is observed that Nano synthesized banana fibre absorbs higher than pure FeS thin films in solar cell application. Finally, green synthesis is an ecofriendly, easy and cheap promising method for the fabrication of thin films for solar cell applications.

## Introduction

Today, ecological challenges such as enlarged air as well as water pollution have prolonged because of populace growth and fast industrialized expansion woeldwide^1,2^. Semiconductors have become the furthermost significant part of investigations throughout the ancient few years, particularly in the areas of electricity and optoelectronic technology^3^. Thin-film technologies are concurrently one of the eldest paintings and one of the latest science associations with thin-film ages to the metallic days of ancient times. Though non-solid and the related matters of interfering insignias have been investigated for over three eras, Integrating thin solid film was perhaps first attained by electrolyses very recently. A composite is a material made up of two or more other materials which give properties, in combination, which is not available from any of the ingredients alone. Nature continues to be generous to mankind by providing all kinds of resources in abundance for his living and existence. In this era of technology, products depend on new varieties of materials that have special characteristics^4,5^. Metal composites, plastic and fibre-reinforced polymer composites are playing a vital role in the fields of nanotechnology. The performance of machine components depends mainly on the material that it is made of in the fields of automobile, railways, aerospace, structural applications, etc., and the strength to weight ratio of the material plays an important role^6^. Due to the improved physical characteristics, the importance of fibers strengthened polymers compounds are gradually substituting numerous of the conservative ingredients^7^. Above the previous era, polymers compounds covered with ordinary fiber have been getting consideration, both from the theoretical biosphere as well as from several manufacturing^8^. Currently, the applications of natural fibers, particularly in motorized manufacturing, have collective repetition. Effectively instigated examples comprise both green fiber thermosets as well as thermo-plastic composite for internal uses such as door boards, shapely parts, and orchestra and tract tables^9^.

Biological technology is the addition of natural technologies as well as engineering to accomplish the claim of creatures, cells, body’s thereof as well as molecules correspondents for yields as well as facilities^10^. Biotechnology is multipurpose and has been considered an important area that has significantly impacted numerous technologies depending on the solicitation of bio procedure in engineering, farming, food-processing, medical approach, eco-friendly protection, and resource upkeep^11^. This new groundswell of high-tech deviations has strong-minded affected enhancements in numerous segments (preparation of medicines, vitamins, minerals, interferon, yields of fermentation serve as nutrition or drink, energy from renewable basis and contaminations and hereditary engineering applied on plants, animals, humans) since it can give completely novel chances for maintainable preparation of surviving and novel yields and services^12^. Additionally, ecological fears help determine the use of biological technology not only for pollution control (decontamination of water, air, and soil) but to preclude pollution and reduce waste in the first place and for ecologically friendly production of chemicals bio-monitoring^12^. The preparation of polycrystalline Iron Sulfide thin films via the biological techniques using enzymes, micro-organisms, and bodies of florae, as well as their excerpts, has been recommended as cheap techniques^13^.

A Nanocomposite material has meaningfully broadened in the previous few years. This term now encompasses a huge diversity of schemes joining one to two as well as three dimensions material with Iron Sulfide constituents variegated at the Nano-meter scales. Natural fibres are universal throughout the world in plants such as flax, sisal, banana, hemp, banana, wood, grasses etc. From naturally available fibres, banana fibre is effortlessly obtainable in fabrics as well as fibres systems with fine mechanically and thermally characteristics^14^.

Cultured banana is resulting from two species of the genus Musa, explicitly from Musa-acuminate and Musa-balbisiana. Musa-acuminate originated from Malaysians, while Musa-balbisiana initiated from Indians. Banana in Africa is categorized into three types, comprising East African(mainly desserts) banana, African plantains bananas grown up largely in the centre as well as west African and the East Africans highlands bananas, applied in cooking and beer preparation^15^. The banana varieties that have been released by the Ethiopian Institute of Agricultural Research (EIAR) are kept in order as (Ducasse Hybrid, Dwarf Cavendish, Giant Cavendish, poyo, Matoke, Nijiru, Kitawira, Cardaba, Butuzua, Robusta, Grand Nain and Williams-1) having Potential Yield (q/ha)^16^ between 261 and 556. Because of availability in the large area, the researchers have done their study on the Williams-1 banana variety.

Banana fibre is biodegradable, cost-effective and lowers compactness fibre with extraordinary precise behaviours. So, banana grounded combination constituents could be applied in industrial, automobile, structural and aerospace applications. Banana fibre is hydrophilic in nature which causes poor wettability with hydrophobic organic matrix resins like polyester when preparing composites^17^. The hydrophobicity nature of banana fibre is reduced by chemical modifications like alkalization, lightening etc. These treatments not only decrease the water absorption capacity of the fibre but also increase the wettability of the fibre with resin and improve inter bond between fibre and matrix. The foremost features of banana fibre are celluloses, lignins, hemicelluloses and pectin^18^. The importance of banana cellulose fibre resulting from yearly renewables resource as a supporting part in polymer atmosphere composite delivers optimistic environmentally welfare with respect to eventual disposable and raw materials. The main advantage of banana cellulose fibers is: a Renewables environment, a wide variety of plasters obtainable throughout the world, Nonfood undeveloped grounded economies, Low-slung energy consumption, cheap, lowest density, Extraordinary precise asset and modulus, great comprehensive repetition of cellulosic based composite, the reprocessing by ignition of cellulose filled composites are easier in contrast with mineral filler systems. The possibility of using banana cellulose fibres as a reinforcing phase has received considerable interest in accumulation, the intrinsic Nanoscale properties of banana fibre cellulose material for developing advanced Nanomaterials and composites^19^.

This environmentally suitable technique for polycrystalline Iron Sulfide (FeS) preparation draws extra significance and is unusual to the biochemical and physical techniques^15^ because of the escaping of the importance of poisonous chemicals as well as maximum energy components in the preparation procedures. Bodies of plant extracts vigorously contribute to the biological decline procedure to change the metallic ions to metals and metallic oxides^16^. Green deposition FeS plays a vital role in contradiction of the degradation procedure of engineering dyes because of photocatalytic influence^20^.

Presently the earth is in fear of airborne and water contamination unconfined from non-renewable energy bases like Coal, natural gases, fossil fuel and gas released from the industry. The excess fluids released out of fabrics pour out to the streams and result in water contamination. This contaminated water is in a straight line drunken by the community and causes diseases such as cholera, amoeba and typhoids. Commonly it disturbs human’s protection fitness in the biosphere. Solar technology connects solar panels, and renewable and Sustainable energy sources^21^. This sustainable energy source starts from natural possessions that endlessly substitute. These contain the sunlight, oceans as well as the power of winds. These types of technologies are considered clean and do not contain carbons since it doesn’t emit greenhouse gas**.** The solar cell is an uncontaminated energy source; it hasn’t an environmental impact on nature like the energy originates from fossil coals. When fossil fuels are burned, it releases hazardous carbon poisonous radiations into the atmosphere^10,22^. To minimize these harmful wastes and contaminations from the environment, the preparation and manufacture of solar energy from compound semiconductors and thin films are the only elucidations. As presently, prevailing elemental semiconductors are very expensive and developing countries like Ethiopia cannot operate them. Widespread vacillating research has been enthusiastic about depositing several types of semiconductor thin films which are used in renewable sources of energy like solar cells^23–25^ due to their applications in the manufacture of photovoltaic ingredients optoelectronic devices, sensors and infrared indicators devices. Iron Sulfide (FeS) thin-film appeals attention of various scholars for they comprise cheap, existence in the large area as well as hold semiconducting materials^26^.In this concern, we have efficiently produced the FeS thin films from *banana* peel extract. The photocatalytic degradation of maximum concentrations of crystal violet has been studied for the first time in facet.

## Materials and methods

**Figure 1:**
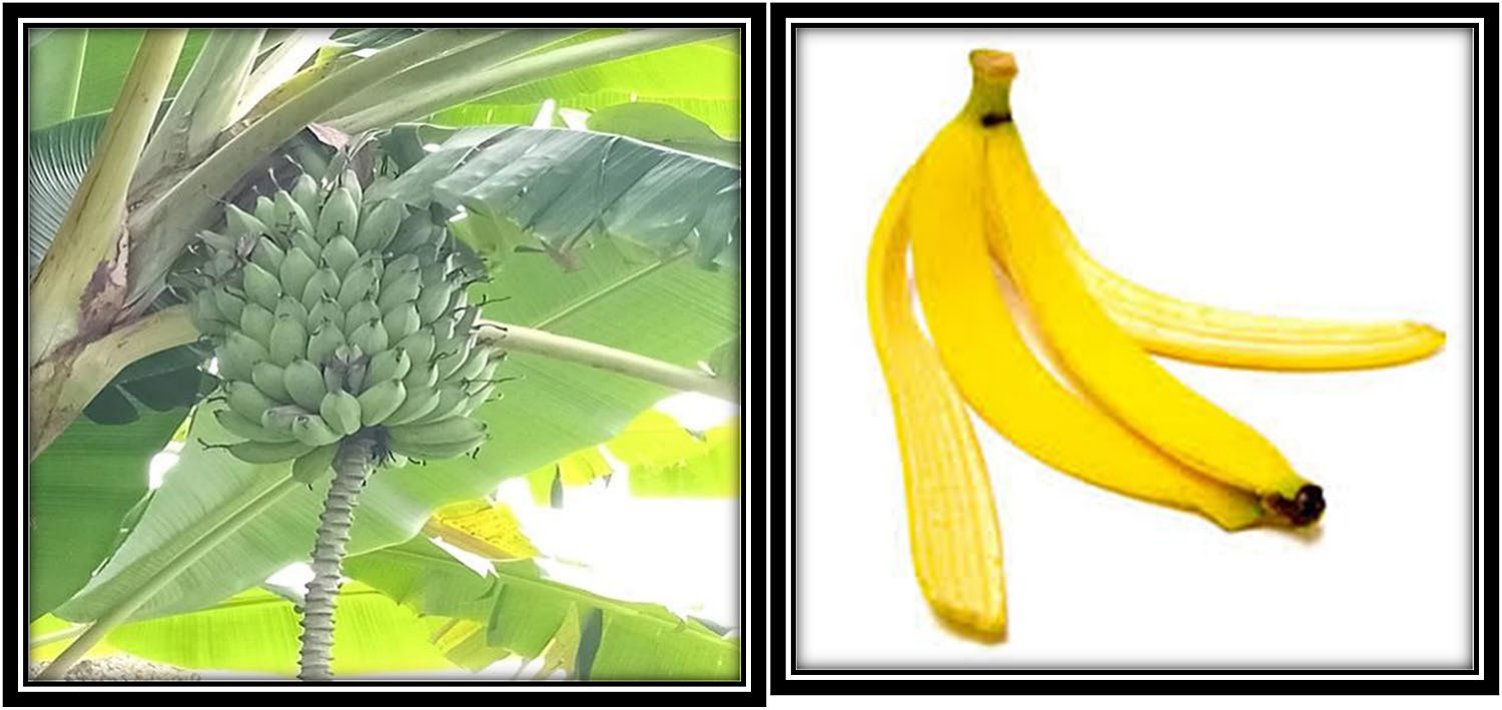
Banana plant with fruit taken from Ethiopian country, Oromia region, Gudaya Bila.

All chemicals, iron chloride, Sodium thiosulfate and Ethylene-di-amine-tetr-acetate (EDTA), were bought from Sigma Aldrich and used without any distillation. Triple deionized water was served as solvents in all laboratory works. The peel of bananas was collected from the local area, Ethiopian country, Oromia region, and Gudaya Bila, as shown in Fig. 1. This peel of banana was polluting the area, and we collected it to make it dry in normal conditions and grind it to make it powder by using a pistol and mortar. The plant we have used in this report was cultivated in Gudaya Bila, Ethiopia. This study complies with relevant legislation and international, national, and institutional guidelines.

### Characterization techniques

**Figure 2:**
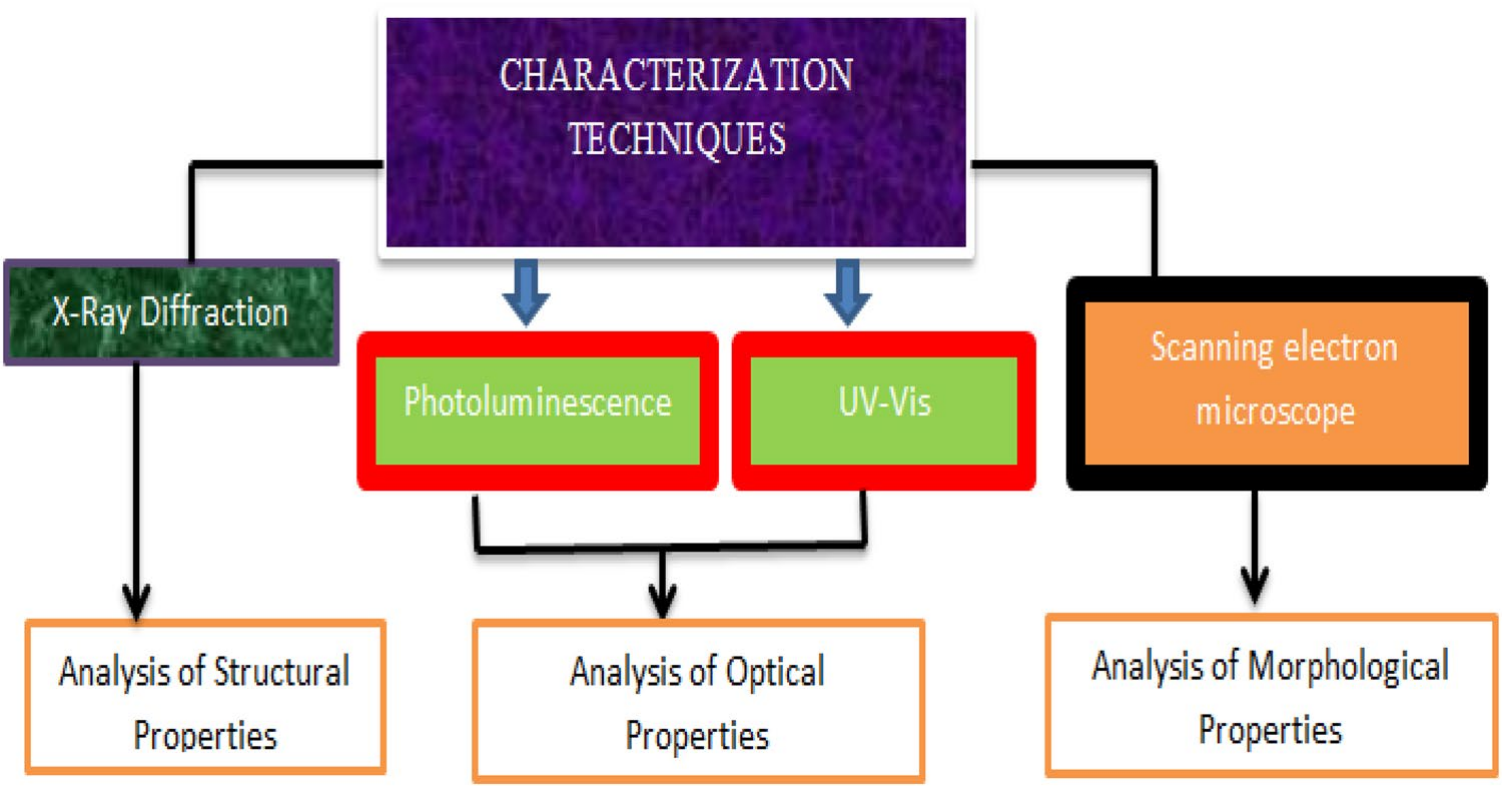
Characterization techniques and their uses.

The structure, morphology and optical characteristics of Iron Sulfide (FeS) were investigated through X-ray diffraction (XRD), Scanning electron microscope(SEM) and UV–vis spectro-photo-meter (UV) and Photoluminescence(PL). Figure 2 displays the characterization techniques with utilities.

Furthermore, the average crystal size of the peel of the banana collected was calculated using the Scherer equation.

D = The crystallite size (D) of the grains can be evaluated using the Scherer’s formula^10^

1 $$ D = \frac{K\lambda }{{\beta Cos\theta }} $$

where K = 0.94 is Scherer constant, $\lambda$ is X-ray wavelength, $\theta$ is Bragg diffraction angle, and $\beta$ is the peak width of the diffraction line at the maximum intensity. Table 1 presents the variation of the measured FeS thin films.

**Table 1:** Crystal structure parameters calculated from XRD graph.

Peaks	2Thetta (degree)	Theta (degree)	FWHM (radian)	Crystal size D (nm)
1	19.76606	9.88	19.76606	0.40797
2	21.37027	10.685	0.89683	9.014491
3	0.09104	0.45	0.63593	12.4924
4	15	7.5	0.7	11.44691

### Experimental details : green deposition of polycrystalline iron sulfide (FeS) using banana peel extraction

The peel of the banana was collected and dried in the normal condition for 3 weeks. Then by using a mortar with a pestle, grinded to get a powder form. In the chemical bath deposition techniques, a water bath beaker with 0.2 M of 250 ml solution was added to 70 mL of Iron Chloride(99.99% purity), 30 mL of Sodium thiosulfate (99.997% purity), and 0.1 M f 10 mL of EDTA (99.89% purity), was added as complexing agent. After appropriate involvement, this solution reaction was placed on a heater of temperature 180℃ with PH value was adjusted to two (PH = 2 acidic bath) adding a droplet of H2SO4 for the 20 min string time and Plastic substrate inserted vertically, then taken at 120 min as sample 1. Then 10 mg of banana peel extract powder was added to the newly prepared solution with the same step. Finally, the substrate was vertically immersed, and the total time of deposition was 120 min (2 h). After the accomplishment of synthesis time, the sample was taken out of the bath as sample 2 and kept in the oven for further characterization.

## Result and discussion

### Structural analysis of polycrystalline FeS thin films

**Figure 3:**
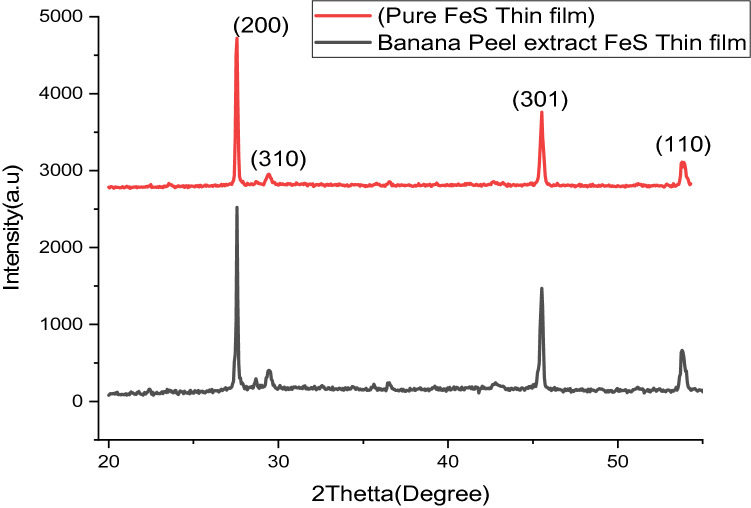
XRD configurations for iron sulfide thin films synthesized as nano-crystalline FeS from banana peel extraction and pure FeS thin films.

Structure characteristics of the organized banana collected were conducted XRD and given in Fig. 3. XRD of FeS prepared by 2.5 mL of 10 mg of banana leaf extract powder was added to the solution (Fig. 3) displays principal crests at 2$\uptheta$ values 12.8°, 28.9°, 37.9°, 46.9°, 50.9°, 69.0°, and 69.9° which are recognized to (110), (200), (310), and (301) Crystal planes with codes COD (R2) = 0.89428054885579, correspondingly. The occurrence of this deflection peak is attributed to the FeS crystal segment of the tetragonal crystalline systems. This result shows in good agreement with nanoparticles published previously^27^.

From XRD patterns, numerous peaks were observed for banana peel extract of FeS thin films and no peak was gained from pure FeS thin films. This shows that the pure FeS thin films are an amorphous structure, and the tetragonal crystal structure was observed for the biosynthesis of FeS thin films from banana peel extract. The crystal size and parameters gained from XRD data are discussed in the table below.

### Optical characterization of iron sulfide (FeS) thin films

**Figure 4:**
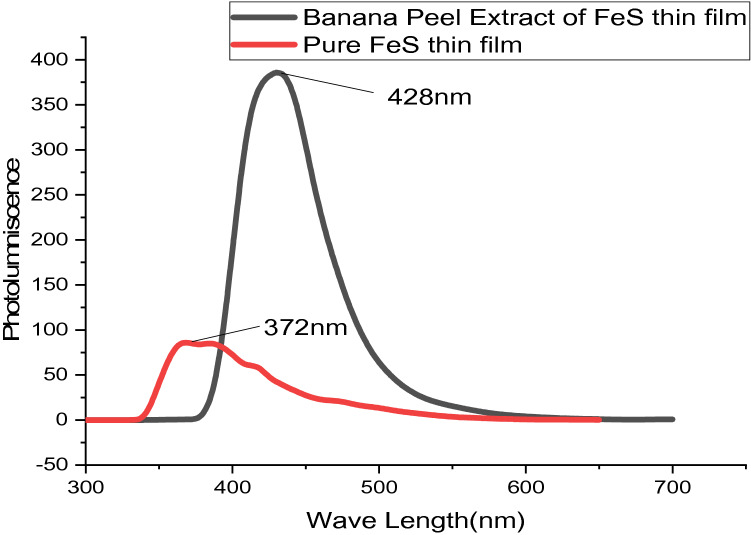
Photoluminescence spectral FeS thin films deposited as nanocrystalline FeS from banana leaf extraction and pure FeS thin films.

**Figure 5:**
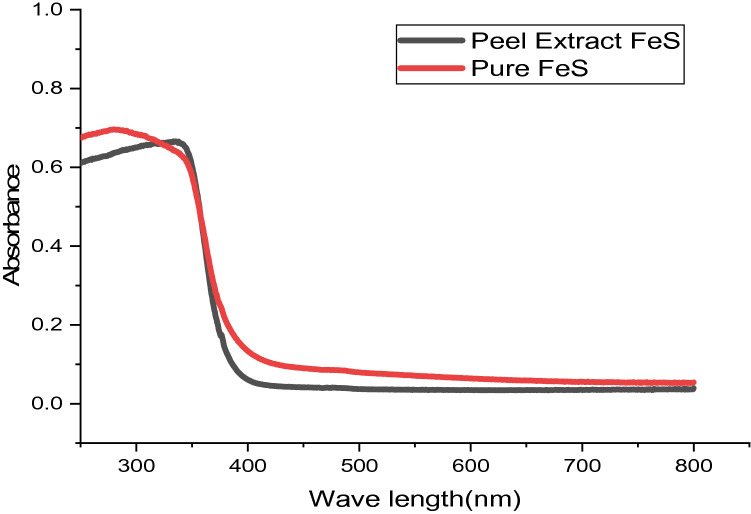
The absorbance of iron sulphide thin films deposited as nanocrystalline FeS from banana leaf extraction and pure FeS thin films.

**Figure 6:**
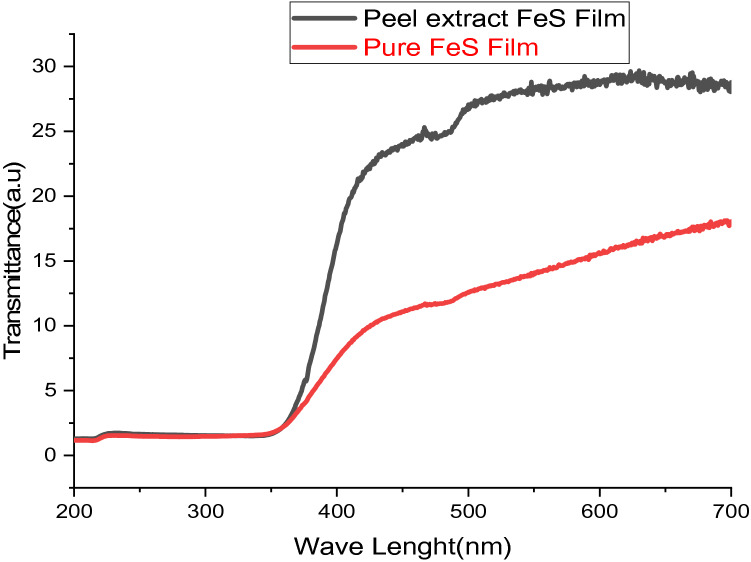
The transmittance of Iron sulphide thin films deposited as nanocrystalline FeS from banana leaf extraction and pure FeS/chemically prepared thin films.

The photoluminescence (PL) spectra of FeS thin films at 180 °C for 2 h prepared using banana peel extracts and pure FeS thin films are displayed in Fig. 4, and spectrums are gained from the U-V; Figs. 5 and 6 show the absorbance and transmittance of excitation wavelength λ = 300–700 nm. It obviously can be seen that the photo-luminescence strength of banana peel extracts FeS thin film is greater than pure FeS thin films in which wide-ranging and symmetries groups were perceived. This may be because the existence of impurity comes from substrates. The profound emanations in the visible range designate the existence of structural defects^28^. A feeble band's emissions in the U-V section are detected at 372–428 nm, conforming to the radiated re-combinations between the animated electron in the conductions band as well as the holes in the valence bands^29–32^. The optical characteristics allow banana peel extracts FeS thin films are useful for photovoltaic solar cells^33^. According to the findings of this research, it is also anticipated to carry out similar studies insight of regulating the shapes of the Nanoparticles and investigating other physical characteristics as reported beforehand on other Nanoscaled metal-sulfide Nanocomposites or oxide^34^.

### Morphological characterization

**Figure 7:**
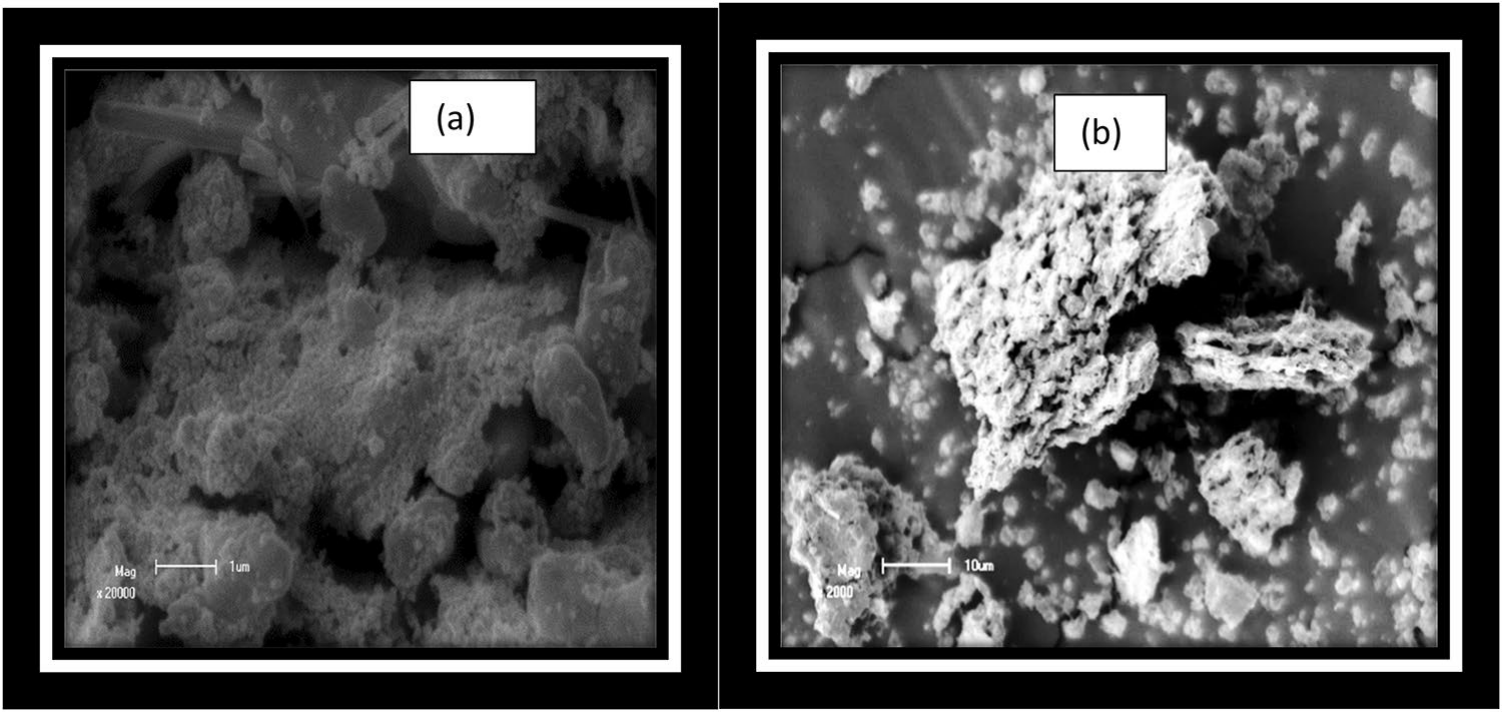
Scanning electron microscope (SEM) FeS thin films deposited as nanocrystalline FeS from banana leaf extraction and pure FeS thin films (**a**,**b**), respectively.

Figure 7 displays the scanning-electron-microscopy (SEM) micrographs of the Iron sulfide films deposited using chemical or pure and banana peel extracts. Depending on Fig. 7a,b, the films prepared by chemical and biological methods confirm complete exposure of materials on superficial substrates. The Scanning electron microscope micrographs of the films prepared from the biological approach show distribution of grains, which covers the surface of the substrate completely and uniform (Fig. 7a). The result may be because of an inadequate quantity of iron source and banana peel extracts of Iron sulfide ions in the solution and has good agreement with reports^35^. The FeS thin film deposited through biological methods is uniformly covered on the substrate. Established on the scanning electron microscope micro-graph, the particle configurations are made on an agglomerate surface (average size is around 21 μ-m)^36^. Comparison between the thin films deposited at biological methods from peel extracts exposes that the amount of Iron Sulfide thin films peak enlarged, demonstrating the best crystal phases for the films prepared^37^. The thin films show slighter grain associated with the other thin films prepared from chemicals only or pure ones (Fig. 7b). The pin-holes are perceived on the superficial of the thin film. This result is finely matched with reports^38–41^.

## Conclusion

Iron Sulfide (FeS) films were effectively deposited through chemical bath deposition techniques. X-Ray Diffraction examination shows the polycrystalline nature of the thin films with the tetragonal segment. Thin films organized through banana peel extract FeS was a higher number of peaks than pure FeS thin films. The superficial morphology of biosynthesized films was detected moderately even and fully deposited on the substrate than the pure FeS thin films. Experimental outcomes showed that the deposition using 0.2 M iron chloride and sodium thiosulphate was the best complaint about the preparation of Iron Sulfide thin films. The spectra are obtained using the UV excitation wavelength λ = 300–700 nm and PL. It is evidently seen that the photo-luminescence strength of banana peel extract FeS thin film is greater than pure FeS thin films in which broads and symmetries bands are witnessed. These profound emanations in the visible ranges show the presence of structural imperfections. Finally, the use of biosynthesis in the production of thin films as a photo absorber is promising for future energy sources which are clean, portable and simple operations.

## Data Availability

The data are included with in the article.
